# Water Extract of *Rubus coreanus* Prevents Inflammatory Skin Diseases In Vitro Models

**DOI:** 10.3390/plants10061230

**Published:** 2021-06-17

**Authors:** Sumin Pyeon, Ok-Kyung Kim, Ho-Geun Yoon, Shintae Kim, Kyung-Chul Choi, Yoo-Hyun Lee, Jeongmin Lee, Jeongjin Park, Woojin Jun

**Affiliations:** 1 Division of Food and Nutrition, Chonnam National University, Gwangju 61186, Korea; sue0567@naver.com (S.P.); 20woskxm@chonnam.ac.kr (O.-K.K.); rotoman1@naver.com (S.K.); 2 Department of Biochemistry and Molecular Biology, College of Medicine, Yonsei University, Seoul 03722, Korea; yhgeun@yuhs.ac.kr; 3 Department of Biomedical Sciences, University of Ulsan College of Medicine, Seoul 05505, Korea; choikc75@amc.seoul.kr; 4 Department of Food and Nutrition, University of Suwon, Hwasung 18323, Korea; creamut@suwon.ac.kr; 5 Department of Medical Nutrition, Kyung Hee University, Yongin 17104, Korea; jlee2007@khu.ac.kr

**Keywords:** atopic dermatitis, *Rubus coreanus*, antioxidant, anti-inflammatory, HaCaT cell

## Abstract

Atopic dermatitis (AD) is a chronic inflammatory skin disease caused by immune hypersensitivity reaction. The cause of AD is unclear, but its symptoms have a negative effect on quality of life; various treatment methods to alleviate these symptoms are underway. In the present study, we aimed to evaluate in vitro antioxidant and anti-inflammatory effects of *Rubus coreanus* water extract (RCW) on AD. Total phenolic compounds and flavonoid content of RCW were 4242.40 ± 54.84 mg GAE/g RCE and 1010.99 ± 14.75 mg CE/g RCW, respectively. RCW reduced intracellular reactive oxygen species level and increased the action of antioxidant enzymes, such as catalase, superoxide dismutase, and glutathione peroxidase in tumor necrosis factor-α (TNF-α)/interferon-γ (IFN-γ)-stimulated HaCaT cells. Moreover, mRNA expression of the pro-inflammatory cytokines, including TNF-α, interleukin-1β, and interleukin-6, was downregulated by RCW in the TNF-α/IFN-γ-stimulated cells. The levels of inflammatory chemokines (thymus- and activation-regulated chemokine; eotaxin; macrophage-derived chemokine; regulated on activation, normal T-cell expressed and secreted; and granulocyte-macrophage colony-stimulating factor) and intercellular adhesion molecule-1 were decreased in the TNF-α/IFN-γ-stimulated HaCaT cells after RCW treatment. Additionally, the mRNA expression levels of filaggrin and involucrin, proteins that form the skin, were increased by RCW. Furthermore, RCW inhibited the nuclear factor kappa-light-chain-enhancer of the activated B cells pathway in the TNF-α/IFN-γ-stimulated HaCaT cells. Collectively, the present investigation indicates that RCW is a potent substance that inhibits AD.

## 1. Introduction

Atopic dermatitis (AD) is a chronic inflammatory skin disease with repeated long-term recovery and recurrence [1]. AD is classified as a type of allergic disease, such as allergic rhinitis and asthma, and presents symptoms such as skin dryness, erythema, edema, pruritus, and desquamation [2]. These symptoms are not life-threatening; however, they not only reduce the quality of life, but are also difficult to treat, as they may occur due to both environmental and genetic factors [3,4]. Although the etiology of AD remains unclear, keratinocytes play a crucial role in being closely associated with excessive T-helper (Th)-2 cells (Th2) immune response [5,6]. Keratinocytes that have initiated an inflammatory response can produce Th2-related chemokines, including thymus- and activation-regulated chemokine (TARC/CCL17); eotaxin (CCL11); macrophage-derived chemokine (MDC/CCL22); and regulated on activation, normal T-cell expressed and secreted (RANTES/CCL5) [7,8]. Moreover, granulocyte-macrophage colony-stimulating factor (GM-CSF) is secreted to differentiate precursor cells such as granulocytes and macrophages and acts as a growth factor for leukocytes. These chemokines prompt the infiltration of immune cells, such as T lymphocytes, monocytes, and mast cells into the skin lesion, thereby inducing inflammatory skin diseases [9]. Additionally, intercellular adhesion molecule-1 (ICAM-1) is excessive expressed in AD skin, causing leukocyte infiltration into the skin [10]. As a result, the proteins that form the skin, such as filaggrin and involucrin, are less expressed, leading to skin spongiosis and edema [11]. Furthermore, the production of pro-inflammatory cytokines, including tumor necrosis factor-α (TNF-α), interleukin-1β (IL-1β), and interleukin-6 (IL-6), is increased in keratinocytes by Th-1 cell (Th1) related cytokines [12].

Further, oxidative stress is induced in the skin in constant contact with oxygen owing to reactive oxygen species (ROS) generation [13]. ROS is produced by the immune system and is used as a defense mechanism against the invasion of foreign substances; however, when it exists in high concentrations in the body, the number of genes expressing inflammatory cytokines increases, thereby causing the inflammatory response to persist and worsen [14]. Thus, patients with AD are particularly sensitive to ROS-induced skin damage [15]. Nevertheless, these patients exhibit antioxidant enzymes such as superoxide dismutase (SOD), catalase (CAT), and glutathione peroxidase (GPx) as a protective mechanism against ROS. Therefore, removal of ROS via activation of antioxidant enzymes is closely associated with alleviation and treatment of AD symptoms [16,17].

Korean blackberry (*Rubus coreanus*, RC) is a perennial deciduous shrub belonging to the Rosaceae family and is a native variety that grows naturally throughout Korea [18]. RC is an affluent source of ascorbic acid and phenolic compounds such as anthocyanin and ellagic acid [19,20]. RC is also well known for its antioxidant activities via radical scavenging, as well as its anti-stress, anti-cancer, and anti-inflammatory effects [21,22]. Recent studies have reported anti-inflammatory effects, in addition to these antioxidant effects. Among the phenolic compounds of RC, anthocyanin and ellagic acid reduce the inflammatory response via the nuclear factor kappa-light-chain-enhancer of activated B cells (NF-κB) pathway [23,24].

In the present study, effects of RC water extract (RCW) on intracellular ROS levels and antioxidant enzyme activity was examined using HaCaT cells. Furthermore, by evaluating changes in the expression of inflammation-related cytokines, atopic dermatitis-related chemokines, and genes related to skin formation proteins, we aimed to confirm the molecular mechanism underlying the anti-inflammatory activity of RCW.

## 2. Materials and Methods

### 2.1. Preparation of RCW

RC used in the experiment was purchased from Jeollanam-do. Dried and milled fruits of RC were extracted by using 20 times of water at 250 °C for 3 h and then extract was filtered (Whatman No. 6; Whatman, Maidstone, UK). The filtered eluate was concentrated using a rotary evaporator in vacuum condition and lyophilized using freeze-dryer. Freeze-dried RCW was stored at −20 °C before use.

### 2.2. Determination of Total Phenolic Compounds and Flavonoid Contents

Total phenolic compounds content of RCW was determined by the Folin–Ciocalteu method of Meda A et al. [25]. RCW was dissolved in deionized water. Dissolved RCW solution (1 mL) was mixed with 9 mL of distilled deionized water (dd H_2_O) and treated 1 mL of Folin-Ciocalteu reagent (Sigma-Aldrich, St. Louis, MO, USA). After reacting in room temperature for 5 min, this solution was mixed with 10 mL of 7% sodium carbonate (Na_2_CO_3_) and 4 mL of dd H_2_O. The mixture was stood at room temperature for 90 min, and the absorbance was measured at 750 nm by a fluorescence microplate reader (BioTek Instruments, Winooski, VT, USA). Gallic acid (GA, Sigma-Aldrich, St. Louis, MO, USA) was used as a standard. The data was expressed as mg GA equivalents (GAE)/100 g lyophilized RCW powder.

Flavonoid content of RCW was determined by the aluminum chloride (AlCl_3_) colorimetric assay [26]. RCW was dissolved in deionized water. Dissolved RCW solution (1 mL) was mixed with 4 mL of dd H_2_O and treated 0.3 mL of 5% sodium nitrate (NaNO_2_). After 6 min in room temperature, 0.3 mL of 5% AlCl_3_, 2 mL of 1 M sodium hydroxide (NaOH), and 2.4 mL dd H_2_O were added. Additionally, the absorbance was then measured at 510 nm by a fluorescence microplate reader. Catechin hydrate (Sigma-Aldrich, St. Louis, MO, USA) was used as a standard. The data was expressed as mg catechin equivalents (CE)/100 g lyophilized RCW powder.

### 2.3. Cell Culture and Treatment

HaCaT cells were obtained from the Cell Line Service (Eppelheim, Germany). The cells were grown in Dulbecco′s minimal essential medium (DMEM; Gibco BRL, Grand Island, NY, USA) supplemented with 10% fetal bovine serum (FBS; Gibco BRL) and 1% penicillin streptomycin (Gibco BRL). Thereafter, the cells were incubated at 37 °C in a humid atmosphere of 5% CO_2_.

### 2.4. Cytotoxicity

HaCaT cells were seeded at a density of 1 × 10^4^ cells/well in a 96-well culture plate and were stabilized at 37 °C for 24 h in an incubator. Subsequently, diverse concentrations of RCW (0–1000 μg/mL) were added to the cells. After treatment for 24 h, the cells were stained using 40 μL of stain reagent, which includes 100 mM phenazine methosulfate (PMS; Sigma-Aldrich) in 1 mg/mL sodium salt of 2,3-bis[2-methoxy-4-nitro-5-sulfophenyl]-2H-tetrazolium-5-carboxyanilide (XTT; Sigma-Aldrich) dissolved in phenol red-free DMEM (PMS:XTT = 1:800). Thereafter, 160 μL of phosphate buffered saline (PBS; Hyclone, Laboratories, Logan, UT, USA) was added to individual wells and incubated for 2 h. Cell viability was estimated using a fluorescence microplate reader with absorbance at 450 nm.

### 2.5. Inducion of Inflammation Using TNF-α/IFN-γ Mixture

HaCaT cells were cultured at a concentration of 1 × 10^5^ cells/well in 6-well culture plates or 2 × 10^4^ cells/well in a 24-well culture plate and incubated for 24 h. Thereafter, they were treated with RCW. Immediately, they were treated simultaneously with TNF-α/IFN-γ mixture (20 ng/mL each) for inducing inflammation.

### 2.6. Assessment of Intracellular ROS Formation

Intracellular ROS levels were assessed using the fluorescence method. HaCaT cells were seeded at a density of 2 × 10^4^ cells/well in a 24-well culture plate and were further incubated for 24 h. Thereafter, the cells were treated with RCW (100 and 200 μg/mL) and TNF-α/IFN-γ mixture for 24 h. Next, the cells were incubated at 37 °C for 30 min while they were exposed to 30 μM 2′7′-dichlorofluorescein diacetate (DCF-DA; Sigma-Aldrich) under light-protected condition. Eventually, the fluorescence intensity of the cells was determined using a fluorescence microplate reader with 485 nm excitation wavelength and 530 nm emission wavelength.

### 2.7. Evaluation of Antioxidant Enzyme Activity

For determining antioxidant enzyme activity, the cells treated with RCW and TNF-α/IFN-γ mixture were lysed using radioimmunoprecipitation assay (RIPA) buffer (Thermo Fisher Scientific, Waltham, MA, USA). The supernatant was obtained via centrifugation at 10,000 rpm at 4 °C for 10 min and was used for the connected assay. The amount of protein was measured via Bradford method using bovine serum albumin (BSA) as the standard reference material. Moreover, CAT activity was evaluated using the spectrophotometric assay based on the decomposition reaction of hydrogen peroxide (H_2_O_2_), as described by Aebi [27]. SOD activity was quantified via microtiter plate assay by Peskin et al. [28], using a water-soluble tetrazolium salt (WST-1; Dojindo, Rockville, MD, USA), xanthine (Sigma-Aldrich), and xanthine oxidase (Sigma-Aldrich). Furthermore, the activity of GPx, which catalyzes the oxidation of GSH to GSSG was determined using the spectrophotometric method of Thomsom et al. [29].

### 2.8. Total RNA Isolation and Real-Time Polymerase Chain Reaction (PCR)

Total RNA was extracted from the RCW and TNF-α/IFN-γ mixture-treated cells using the easy-BLUE™ total RNA extraction kit (Intron Biotechnology, Seongnam, Korea), according to manufacturer’s instructions. Thereafter, complementary DNA (cDNA) was synthesized using the iScript cDNA Synthesis Kit (Bio-Rad Laboratories, Hercules, CA, USA) with 150 ng of purified total RNA. Real-time PCR was carried out using the SYBR green Real-time PCR kit (Bio-Rad) with 1 μL synthesized cDNA. Next, cDNA was amplified with 40 cycles of denaturation (95 °C for 30 s), annealing (55 °C for 30 s), and extension (72 °C for 60 s), using custom-designed primers (Table 1).

### 2.9. Western Blotting

After treatment with the stimulant and sample, the cells washed with PBS were lysed for 20 min with RIPA buffer containing protease inhibitor and phosphatase inhibitor. The supernatant collected after centrifugation was quantified via Bradford assay to determine the amount of protein, and each sample containing 30 μg of protein was loaded on 10% sodium dodecyl sulfonate-polyacrylamide gel electrophoresis (SDS-PAGE) for separation. Next, after transferring the whole protein to the polyvinylidene fluoride (PVDF) membrane, it was blocked with 3% BSA in PBS for 1 h. Thereafter, the primary antibodies diluted in 0.5% BSA in PBS at 1:1000 were treated and reacted at 4 °C overnight. After washing thrice with tris-buffered saline (TBS) containing 0.1% Tween 20 (TBST), the secondary antibody diluted in 0.5% BSA dissolved in PBS at 1:5000 was treated for 1 h. In order to identify the target protein, the enhanced chemiluminescence (ECL) substrate (Bio-Rad, Rad Laboratories, Hercules, CA, USA) was reacted for 1 min. The target protein band was observed and photographed by Chemidoc XRS+ (Bio-Rad, Rad Laboratories, Hercules, CA, USA).

### 2.10. Statistical Analysis

Data are presented as mean ± standard deviation (S.D.). The data were statistically analyzed by applying one-way analysis of variance (ANOVA) using SPSS statistical procedures for Windows (SPSS PASW Statistics 25.0; SPSS Inc., Chicago, IL, USA). In this case, significant differences between groups were compared with Duncan′s multiple range test (*p* < 0.05). Furthermore, to compare the significant differences between two groups, Student′s t-test were used (*p* < 0.05).

## 3. Results

### 3.1. Total Phenolic Compounds and Flavonoid Content of RCW

The results showed that the total phenolic compounds and flavonoid content in the RCW. Total phenolic compounds content was 4242.40 ± 54.84 mg GAE/100 g RCW and flavonoid content was 1010.99 ± 14.75 mg CE/100 g RCW (Table 2). According to the results of our study, RCW appears to have enough antioxidants. Therefore, we tried to study the effect of RCW on inflammatory skin disease.

### 3.2. Cytotoxicity of RCW in HaCaT Cells

**Figure 1:**
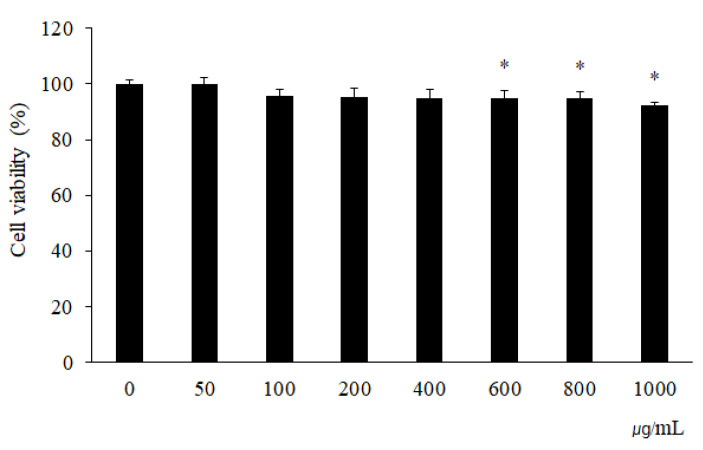
Cell viability of RCW in HaCaT cells. Data express the mean ± S.D. The asterisks above the bar indicate significant difference from the 0 μg/mL group using Student’s *t*-test (*p* < 0.05).

To know the safety range, the cytotoxicity of RCW in HaCaT cells was assessed by treating the cells with diverse concentrations of RCW (0–1000 μg/mL). The cell viability was remarkably decreased at a concentration of 600 μg/mL (Figure 1). Then, 100–200 μg/mL RCW, representing the nontoxic range, was used in further study for assessing its antioxidant and AD inhibitory effect.

### 3.3. Effect of RCW on Intracellular ROS Level in TNF-α/IFN-γ-Stimulated HaCaT Cells

**Figure 2:**
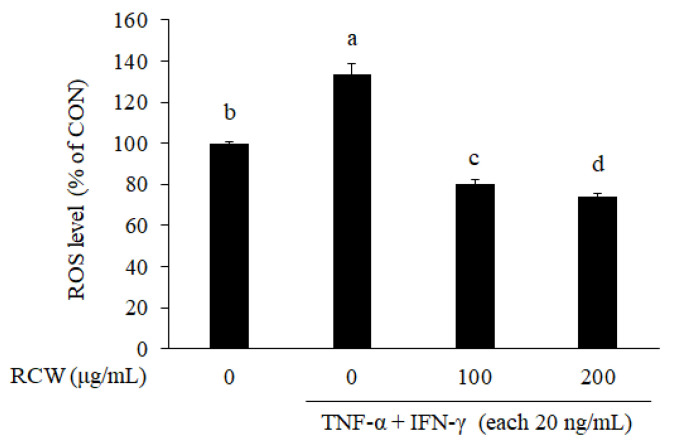
Effect of RCW on intracellular ROS level in TNF-α/IFN-γ-stimulated HaCaT cells. Data express the mean ± S.D. CON, non-stimulated HaCaT cells; T/I, TNF-α/IFN-γ-stimulated HaCaT cells; RCW100, treatment of 100 μg/mL RCW in TNF-α/IFN-γ-stimulated HaCaT cells; RCW200, treatment of 200 μg/mL RCW in TNF-α/IFN-γ-stimulated HaCaT cells. The different letters above the bar indicate significant difference between groups using the Duncan′s multiple range test (*p* < 0.05).

Excessive ROS in the body is interrelated to the occurrence of inflammatory response [14]. The intracellular ROS level of the TNF-α/IFN-γ-stimulated HaCaT cells was prominently higher than that of the control. When the TNF-α/IFN-γ-stimulated HaCaT cells were treated with 100 and 200 μg/mL RCW, the intracellular ROS level decreased in a dose-dependent manner (Figure 2). This result suggests that RCW has the ability to remove intracellular ROS in TNF-α/IFN-γ-stimulated HaCaT cells.

### 3.4. Effects of RCW on Antioxidant Enzyme Activity in TNF-α/IFN-γ-Stimulated HaCaT Cells

The activity of antioxidant enzyme was significantly lower in the TNF-α/IFN-γ-treated cells than in the control; however, this activity was significantly recovered when TNF-α/IFN-γ in HaCaT cells were treated with RCW. In particular, Gpx activity attained a similar level as that of control when treated with 200 μg/mL RCW (Table 3). The results suggest that RCW exhibits antioxidant activity in AD-like state-simulated HaCaT cells.

### 3.5. Effects of RCW on Proinflammatory Cytokines in TNF-α/IFN-γ-Stimulated HaCaT Cells

**Figure 3:**
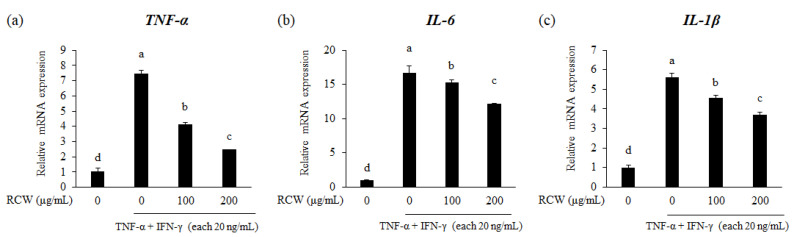
Effects of RCW on gene expression of proinflammatory cytokines in TNF-α/IFN-γ-stimulated HaCaT cells. Relative expression levels of all genes were examined using real-time PCR. *GAPDH* was used as the internal control to standardize the expression levels in each sample. (**a**) Relative mRNA expression level of *TNF-α*. (**b**) Relative mRNA expression level of *IL-6*. (**c**) Relative mRNA expression level of *IL-1β*. Data express the mean ± S.D. The different letters above the bar indicate significant difference between groups using the Duncan′s multiple range test (*p* < 0.05).

Proinflammatory cytokines including *TNF-α*, *IL-6*, and *IL-1β* promote systemic inflammatory responses [30]. The TNF-α/IFN-γ-stimulated HaCaT cells showed a remarkable increase in the mRNA expression levels of *TNF-α*, *IL-6*, and *IL-1β*, compared to those in TNF-α/IFN-γ-nontreated HaCaT cells. When TNF-α/IFN-γ-stimulated HaCaT cells were treated with RCW, the mRNA expression levels of *TNF-α*, *IL-6*, and *IL-1β* decreased in a dose dependent manner (Figure 3). These results suggest that RCW efficiently reduces inflammation.

### 3.6. Effects of RCW on Chemokines and Adhesion Molecule in TNF-α/IFN-γ-Stimulated HaCaT Cells

**Figure 4:**
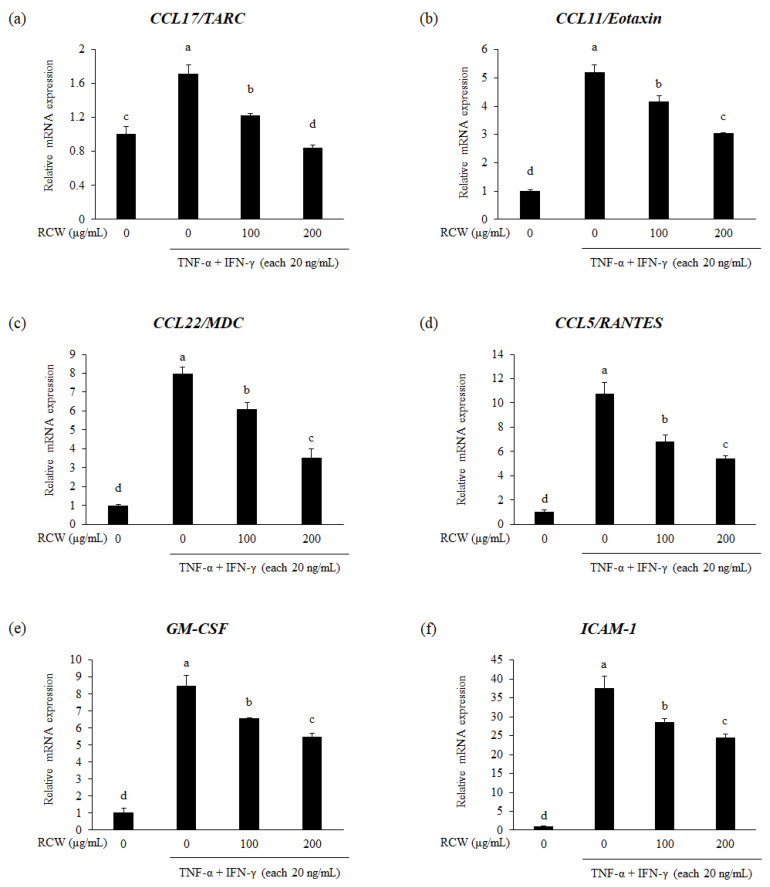
Effects of RCW on gene expression of inflammatory chemokines and adhesion molecule in HaCaT cells. Relative expression levels of all genes were verified using real-time PCR. *GAPDH* was used as the internal control for standardizing expression levels in each sample. (**a**) Relative mRNA expression level of *CCL17/TARC*. (**b**) Relative mRNA expression level of *CCL11/Eotaxin*. (**c**) Relative mRNA expression level of *CCL22/ MDC*. (**d**) Relative mRNA expression level of *CCL5/RANTES*. (**e**) Relative mRNA expression level of *GM-CSF*. (**f**) Relative mRNA expression level of *ICAM-1*. Data express the mean ± S.D. The different letters above the bar indicate significant difference between groups using the Duncan′s multiple range test (*p* < 0.05).

The mRNA expression levels of inflammatory chemokines, such as *CCL17/TARC*, *CCL11/Eotaxin*, *CCL22/MDC*, *CCL5/RANTES*, and *GM-CSF*, were significantly increased in TNF-α/IFN-γ-stimulated HaCaT cells. The group simultaneously treated with TNF-α/IFN-γ mixture and RCW revealed that the mRNA expression level of chemokines reduces in a dose-dependent manner; however, a significant decrease was observed when compared to that in the group treated with only TNF-α/IFN-γ mixture. Moreover, the expression level of *ICAM-1*, an adhesion molecule, was increased in the presence of cytokines such as TNF-α and IL-6, after treatment with TNF-α/IFN-γ in HaCaT cells [31]; however, it was decreased when these TNF-α/IFN-γ-stimulated HaCaT cells were treated with RCW (Figure 4).

### 3.7. Effects of RCW on Expression of Genes Related to Skin Formation in TNF-α/IFN-γ-Stimulated HaCaT Cells

**Figure 5:**
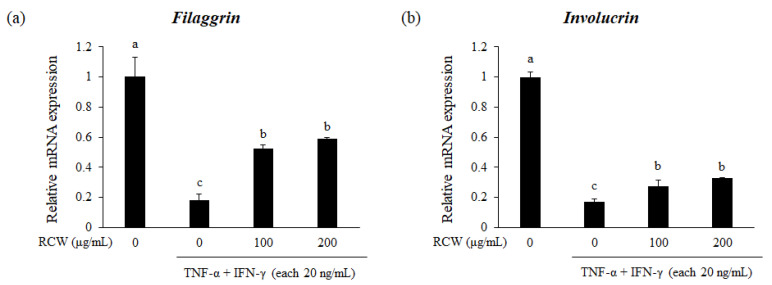
Effects of RCW on expression of genes related to skin formation in HaCaT cells. Relative expression of all genes were verified using real-time PCR. *GAPDH* was used as the internal control for standardizing expression levels in each sample. (**a**) Relative mRNA expression level of filaggrin. (**b**) Relative mRNA expression level of involucrin. Data express the mean ± S.D. The different letters above the bar indicate significant difference between groups using the Duncan′s multiple range test (*p* < 0.05).

The mRNA expression levels of genes related to skin formation, including filaggrin and involucrin, were declined in TNF-α/IFN-γ-stimulated HaCaT cells. The aforementioned results occur because these proteins constitute the skin barrier and protect themselves from various external stimuli; hence, in AD, the skin barrier is disrupted and leads to inflammatory reaction [32]. In contrast, the expression levels of these genes were notably increased in RCW-treated groups (Figure 5), thereby indicating that RCW restores the skin barrier damaged by AD.

### 3.8. Effects of RCW on the NF-κB Pathway in TNF-α/IFN-γ-Stimulated HaCaT Cells

**Figure 6:**
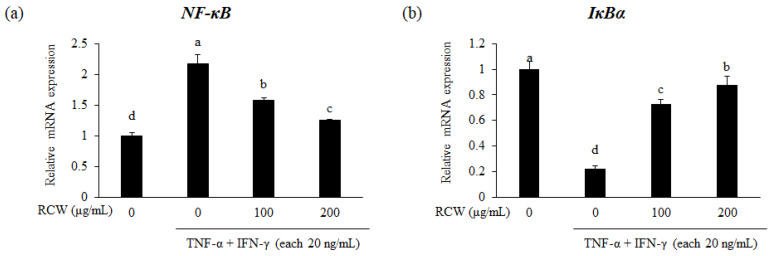
Effects of RCW on expression of *NF-κB* and *IκBα* genes in HaCaT cells. Relative expression levels of all genes were verified using real-time PCR. *GAPDH* was used as the internal control for standardizing expression levels in each sample. (**a**) Relative mRNA expression level of *NF-κB*. (**b**) Relative mRNA expression level of *IκBα*. Data express the mean ± S.D. The different letters above the bar indicate significant difference between groups using the Duncan′s multiple range test (*p* < 0.05).

The NF-κB signaling pathway regulates inflammatory factors such as IL-6, TARC, MDC, and RANTES in HaCaT cells [33,34]. When TNF-α/IFN-γ was treated in HaCaT cells, increased gene expression level was observed for *NF-κB*. In contrast, inhibitor of NF-κB alpha (*IκBα*) gene expression level was decreased in TNF-α/IFN-γ-stimulated HaCaT cells; however, RCW exerted opposite effect (Figure 6). Therefore, these results indicate that the anti-inflammatory effect of RCW on AD is mediated via the NF-κB signaling pathway.

### 3.9. Effects of RCW on the NF-κB Pathway in TNF-α/IFN-γ-Stimulated HaCaT Cells

**Figure 7:**
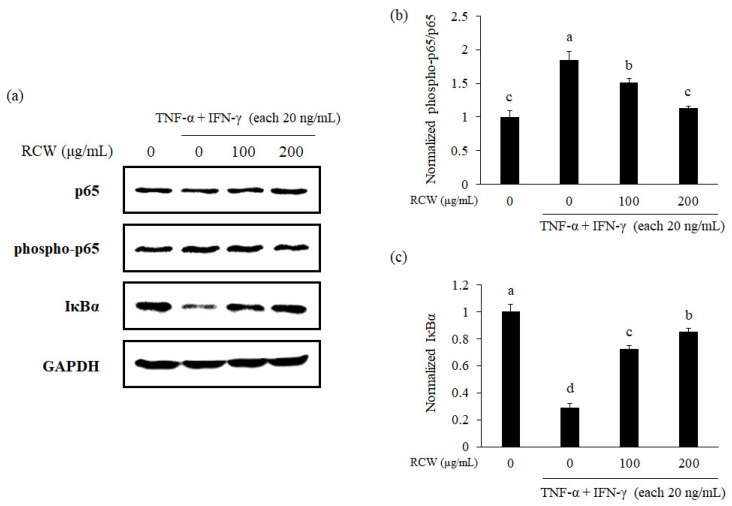
Effects of RCW on protein levels of NF-κB and IκBα in HaCaT cells. Relative expression of all genes was determined by Western blotting. GAPDH was used as the internal control for standardizing the expression levels in each sample. (**a**) Representative Western blots of p65, phospho-p65, IκBα, and GAPDH. (**b**) Normalized protein level of phospho-p65/p65. (**c**) Normalized protein level of IκBα. Data express the mean ± S.D. The different letters above the bar indicate significantly difference between groups using the Duncan′s multiple range test (*p* < 0.05).

Based on the previous results, we performed Western blot to quantify the expression of NF-κB p65, its phosphorylation form and IκBα. When TNF-α/IFN-γ mixture was added to HaCaT cells, the phosphorylated form of NF-kB p65 increased; however, it was decreased in a concentration-dependent manner by RCW. In contrast, IκBα level decreased in TNF-α/IFN-γ-stimulate HaCaT cells, but increased in a concentration-dependent manner after RCW treatment (Figure 7).

## 4. Discussion

In this study, we evaluated the antioxidant and anti-inflammatory effects of RCW on TNF-α/IFN-γ-stimulated HaCaT cells, which are human-derived keratinocytes. RCW contains 4242.40 ± 54.84 mg GAE/100 g RCW total phenolic compounds and 1010.99 ± 14.75 mg CE/100 g RCW flavonoids. Therefore, it is confirmed as a material that is rich in bioactive compounds capable of antioxidant activity. Keratinocytes protect the outer barrier of epidermis using their own immune responses, including secretion of inflammatory cytokines and induction of immune cell infiltration [35,36]. As reported in previous studies, treatment with TNF-α/IFN-γ mixture triggers the aforementioned immune response, because TNF-α is known to promote immunity and trigger inflammation in chronic diseases; moreover, IFN-γ is a cytokine that plays a pivotal role in inflammation and immune response in the skin and regulates the activity of various cells [37,38]. This suggests that reducing excessive immune responses by targeting TNF-α and IFN-γ can aid in the treatment of AD.

Oxidative stress caused by excess ROS damages the skin barrier and allows the inflammatory response to persist [15]. This occurs because AD is caused by an immune hypersensitivity reaction [39]. Therefore, ROS reduction in patients with AD has remarkable effects on the relief and recovery of symptoms. We confirmed that the intracellular ROS increased significantly when TNF-α and IFN-γ were added to HaCaT cells; however, it decreased in a dose-dependent manner when these TNF-α/IFN-γ stimulated cells were treated with RCW (Figure 2). Moreover, antioxidant enzymes, such as SOD, CAT, and GPx, can reduce oxidative stress by removing ROS from the body, thereby relieving the AD symptoms [16,17]. From these results, it was confirmed that RCW exhibits effective antioxidant activity in TNF-α/IFN-γ-stimulated keratinocytes.

Keratinocytes stimulated by TNF-α and IFN-γ secrete the inflammatory cytokines TNF-α, IL-6, and IL-1β, which further increase the expression level of the chemokines (CCL17/TARC, CCL11/Eotaxin, CCL22/MDC, CCL5/RANTES, and GM-CSF) and the adhesion molecule, *ICAM-1* (Figure 3 and Figure 4). CCL17/TARC is a chemokine that deposits T cells in the epidermis, causes apoptosis of keratinocytes, and induces spongiosis of the skin [40]. Furthermore, chemokines affect the accumulation of various immune cells in the skin tissue, such as CCL11/Eotaxin to eosinophils; CCL22/MDC to monocytes and natural killer (NK) cells; and CCL5/RANTES to eosinophils, mast cells and monocytes [41,42,43,44]. GM-CSF promotes the differentiation and maturation of progenitor cells such as granulocytes and macrophages, which is associated with chronic AD and severe inflammation [44]. ICAM-1 is a ligand for receptors of leukocytes and is involved in leukocyte penetration during inflammatory reactions [10,45].

In patients with AD, the skin barrier is damaged due to the skin inflammatory reaction promoted by the infiltration of the allergen and decrease in the expression levels of filaggrin and involucrin, which are crucial components of stratum corneum in the skin [11,46]. Filaggrin and involucrin are present in the keratinocyte membrane and allow aggregation of keratin fibers and binding of structural proteins, thereby resulting in a hard and flat structure [47]. Their reduction decreases the adhesion between keratinocytes and increases transepidermal water loss, thereby reducing overall skin barrier function, and increasing the penetration of allergens, and facilitating allergic reactions [48]. Moreover, pyrrolidone carboxylic acid and urocanic acid, which are separated units from filaggrin, are natural moisturizing factors that help to hydrate the stratum corneum, normalize pH, and have antibacterial and anti-inflammatory effects [49,50]. Therefore, recovery of filaggrin and involucrin, which are reduced in AD conditions, may help in relieving symptoms such as skin dryness, erythema, psoriasis, and desquamation.

The NF-κB signaling pathway triggers the inflammatory responses in TNF-α/IFN-γ-stimulated keratinocytes [51]. This pathway is known to regulate the expression of TNF- α, IL-1β, IL-6, GM-CSF, CCL5/RANTES, CCL11/Eotaxin, and ICAM-1 in AD [52,53]. We demonstrated that the expression of NF-κB was downregulated by RCW in TNF-α/IFN-γ-stimulated keratinocytes. Conversely, the expression level of IκBα, which inhibits the activity of NF-κB, was increased by RCW treatment (Figure 6). Hence, it was confirmed that the NF-κB signaling pathway in HaCaT cells is inhibited by RCW to improve AD.

## 5. Conclusions

The present study shows that RCW relieves AD symptoms in an in vitro model (TNF-α/IFN-γ-stimulated HaCaT cells) by exerting antioxidant effects; suppressing inflammatory mediators; and increasing the levels of proteins that form the skin, via regulating the NF-κB signaling pathway. Therefore, these results suggest that RCW has potential for the treatment or prevention of inflammatory skin diseases, including AD.

## Figures and Tables

**Table 1:** Sequence of real-time PCR primers.

Gene	Primer Sequence (5′ to 3′)	
*TNF-α*	Forward	5′- CCACTTCGAAACCTGGGATTC-3′
	Reverse	5-′TTAGTGGTTGCCAGCACTTCA-3′
*IL-6*	Forward	5′-AGGGCTCTTCGGCAAATGTA-3′
	Reverse	5′-GAAGGAATGCCCATTAACAACAA-3′
*IL-1β*	Forward	5′-GCATCCAGCTACGAATCTCC-3′
	Reverse	5′-GGAACCAGCATCTTCCTCAG-3′
*CCL17/TARC*	Forward	5′-GAAGACGTGGTACCAGACATCTGA-3′
	Reverse	5′-CCCTGCACAGTTACAAAAACGA-3′
*CCL11/Eotaxin*	Forward	5′-GCGACTAGAGAGCTACAGGAGAATC-3′
	Reverse	5′-GGTCTTGAAGATCACAGCTTTCTG-3′
*CCL22/MDC*	Forward	5′-GTTGTCCTCGTCCTCCTTGC-3′
	Reverse	5′-GGAGTCTGAGGTCCAGTAGAAGTG-3′
*CCL5/RANTES*	Forward	5′-AGTGTGTGCCAACCCAGAGA-3′
	Reverse	5′-AGCAAGCAGAAACAGGCAAA-3′
*GM-CSF*	Forward	5′-ACTTCCTGTGCAACCCAGATT-3′
	Reverse	5′-CATCTGGCCGGTCTCACTC-3′
*ICAM-1*	Forward	5′-CAAGGCCTCAGTCAGTGTGA-3′
	Reverse	5′-CCTCTGGCTTCGTCAGAATC-3′
*Filaggrin*	Forward	5′-GCAAGGTCAAGTCCAGGAGAA-3′
	Reverse	5′-CCCTCGGTTTCCACTGTCTC-3′
*Involucrin*	Forward	5′-CTGCCTGAGCAAGAATGTGA-3′
	Reverse	5′-AGCTGCTGATCCCTTTGTGT-3′
*NF-κB*	Forward	5′-CTCCTGTGCGTGTCTCCATG-3′
	Reverse	5′-TTACGTTTCTCCTCAATCCG-3′
*IκBα*	Forward	5′-GAAATACCCCCCTACACCTT-3′
	Reverse	5′-GACACCAAAAGCTCCACGAT-3′
*GAPDH*	Forward	5′-CTGCTCCTCCTGTTCGACAGT-3′
	Reverse	5′-CCGTTGACTCCGACCTTCAC-3′

**Table 2:** Total phenolic compounds and flavonoid content of RCW.

	Total Phenolic Compounds Content (mg GAE/100 g RCW)	Flavonoid Content (mg CE/100 g RCW)
RCW	4242.40 ± 54.84	1010.99 ± 14.75

**Table 3:** Effects of RCW on antioxidant enzyme activity in TNF-α/IFN-γ-stimulated HaCaT cells.

	CAT (U/mg Protein)	SOD (U/mg Protein)	GPx (U/mg Protein)
CON	4.48 ± 0.66 ^a^	62.35 ± 2.94 ^a^	0.0102 ± 0.0001 ^a^
T/I	2.02 ± 0.08 ^c^	34.70 ± 2.23 ^d^	0.0058 ± 0.0001 ^c^
RCW 100	2.20 ± 0.17 ^c^	39.28 ± 1.85 ^c^	0.0075 ± 0.0002 ^b^
RCW 200	2.92 ± 0.27 ^b^	50.72 ± 1.09 ^b^	0.0099 ± 0.0004 ^a^

CON, non-stimulated HaCaT cells; T/I, TNF-α/IFN-γ-stimulated HaCaT cells; RCW100, treatment of 100 μg/mL RCW in the TNF-α/IFN-γ-stimulated HaCaT cells; RCW200, treatment of 200 μg/mL RCW in the TNF-α/IFN-γ-stimulated HaCaT cells. Data express the mean ± S.D. The different letters above the bar indicate significant differences between groups using the Duncan′s multiple range test (*p* < 0.05).

## References

[1] Wallach D., Taïeb A. (2014). Atopic dermatitis/atopic eczema. Chem. Immunol. Allergy.

[2] Suárez-Fariñas M., Tintle S.J., Shemer A., Chiricozzi A., Nograles K., Cardinale L., Duan S., Bowcock A.M., Krueger J.G., Guttman-Yassky E. (2011). Nonlesional atopic dermatitis skin is characterized by broad terminal differentiation defects and variable immune abnormalities. J. Allergy Clin. Immunol..

[3] Wollenberg A., Kraft S., Oppel T., Bieber T. (2000). Atopic dermatitis: Pathogenetic mechanisms. Clin. Exp. Dermatol..

[4] McGrath J.A. (2008). Filaggrin and the great epidermal barrier grief. Australas. J. Dermatol..

[5] Ong P.Y., Leung D.Y.M. (2006). Immune dysregulation in atopic dermatitis. Curr. Allergy Asthma Rep..

[6] Giustizieri M.L., Mascia F., Frezzolini A., De Pità O., Chinni L.M., Giannetti A., Girolomomi G., Pastore S. (2001). Keratinocytes from patients with atopic dermatitis and psoriasis show a distinct chemokine production profile in response to T cell-derived cytokines. J. Allergy Clin. Immunol..

[7] Jahnz-Rozyk K., Targowski T., Paluchowska E., Owczarek W., Kucharczyk A. (2005). Serum thymus and activation-regulated chemokine, macrophage-derived chemokine and eotaxin as markers of severity of atopic dermatitis. Allergy Eur. J. Allergy Clin. Immunol..

[8] Lee K.S., Chun S.Y., Lee M.G., Kim S., Jang T.J., Nam K.S. (2018). The prevention of TNF-α/IFN-γ mixture-induced inflammation in human keratinocyte and atopic dermatitis-like skin lesions in Nc/Nga mice by mineral-balanced deep sea water. Biomed. Pharmacother..

[9] Kim J.R., Choi J., Kim J., Kim H., Kang H., Kim E.H., Chang J.H., Kim Y.E., Choi Y.J., Lee K.W. (2014). 20-O-β-d-glucopyranosyl-20(S)-protopanaxadiol-fortified ginseng extract attenuates the development of atopic dermatitis-like symptoms in NC/Nga mice. J. Ethnopharmacol..

[10] Jung K., Linse F., Heller R., Moths C., Goebel R., Neumann C. (1996). Adhesion molecules in atopic dermatitis: VCAM-1 and ICAM-1 expression is increased in healthy-appearing skin. Allergy Eur. J. Allergy Clin. Immunol..

[11] Katzka D.A., Tadi R., Symyrk T.C., Katarya E., Sharma A., Geno D.M., Camilleri M., Lyer P.G., Alexander J.A., Buttar N.S. (2014). Effects of topical steroids on tight junction proteins and spongiosis in esophageal epithelia of patients with eosinophilic esophagitis. Clin. Gastroenterol. Hepatol..

[12] Lee H.S., Choi E.J., Choi H., Lee K.S., Kim H.R., Na B.R., Kwon M.S., Jeong G.S., Choi H.G., Choi E.Y. (2015). Oral administration of 4-hydroxy-3-methoxycinnamaldehyde attenuates atopic dermatitis by inhibiting T cell and keratinocyte activation. PLoS ONE.

[13] Ji H., Li X.K. (2016). Oxidative stress in atopic dermatitis. Oxid. Med. Cell. Longev..

[14] Valko M., Leibfritz D., Moncol J., Cronin M.T.D., Mazur M., Telser J. (2007). Free radicals and antioxidants in normal physiological functions and human disease. Int. J. Biochem. Cell Biol..

[15] Sivaranjani N., Venkata Rao S., Rajeev G. (2013). Role of reactive oxygen species and antioxidants in atopic dermatitis. J. Clin. Diagn. Res..

[16] Mates J.M., Perez-Gomez C., De Castro I.N. (2015). Antioxidant enzymes and human disease. IOP Conf. Ser. Mater. Sci. Eng..

[17] Kiyohara C., Tanaka K., Miyake Y. (2008). Genetic susceptibility to atopic dermatitis. Allergol. Int..

[18] Lim J.W., Jeong J.T., Shin C.S. (2012). Component analysis and sensory evaluation of Korean black raspberry (Rubus coreanus Mique) wines. Int. J. Food Sci. Technol..

[19] Lee Y.A., Lee M.W. (1995). Tannins from Rubus coreanum. Korean J. Pharmacogn..

[20] Park Y.K., Choi S.H., Kim S.H., Jang Y.S., Han J., Chung H.G. (2008). Functional composition and antioxidant activity from the fruits of Rubus coreanus according to cultivars. J. Korean Wood Sci. Technol..

[21] Yang H.M., Lim S.S., Lee Y.S., Shin H.K., Oh Y.S., Kim J.K. (2007). Comparison of the anti-inflammatory effects of the extracts from Rubus coreanus and Rubus occidentalis. Korean J. Food Sci. Technol..

[22] Kim J.H., Kim C.H., Kim H.S., Kwon M.C., Song Y.K., Seong N.S., Lee S.E., Yi J.S., Kwon O.W., Lee H.Y. (2006). Effect of Aqueous Extracts from Rubus coreanus Miquel and Angelica gigas Nakai on Anti-tumor and Anti-stress activities in mice. Korean J. Med. Crop Sci..

[23] Lim J.W., Hwang H.J., Shin C.S. (2012). Polyphenol compounds and anti-inflammatory activities of Korean black raspberry (Rubus coreanus Miquel) wines produced from juice supplemented with pulp and seed. J. Agric. Food Chem..

[24] Seo K.H., Lee J.Y., Park J.Y., Jang G.Y., Kim H.D., Lee Y.S., Kim D.H. (2019). Differences in anti-inflammatory effect of immature and mature of Rubus coreanus fruits on LPS-induced RAW 264.7 macrophages via NF-κB signal pathways. BMC Complement. Altern. Med..

[25] Meda A., Lamien C.E., Romito M., Millogo J., Nacoulma O.G. (2005). Determination of the total phenolic, flavonoid and proline contents in Burkina Fasan honey, as well as their radical scavenging activity. Food Chem..

[26] Atanassova M., Georgieva S., Ivancheva K. (2011). Total phenolic and total flavonoid contents, antioxidant capacity and biological contaminants in medicinal herbs. J. Univ. Chem. Technol. Metall..

[27] Aebi H. (1974). Catalase. Methods Enzym. Anal..

[28] Peskin A.V., Winterbourn C.C. (2017). Assay of superoxide dismutase activity in a plate assay using WST-1. Free Radic. Biol. Med..

[29] Thomson C.D., Rea H.M., Robinson M.F., Simpson F.O. (1977). Selenium concentrations and glutathione peroxidase activities in blood of hypertensive patients. Br. J. Nutr..

[30] Dinarello C.A. (2000). Proinflammatory cytokines. Chest.

[31] Hubbard A.K., Rothlein R. (2000). Intercellular adhesion molecule-1 (ICAM-1) expression and cell signaling cascades. Free Radic. Biol. Med..

[32] Furue M. (2020). Regulation of filaggrin, loricrin, and involucrin by IL-4, IL-13, IL-17A, IL-22, AHR, and NRF2: Pathogenic implications in atopic dermatitis. Int. J. Mol. Sci..

[33] Choi J.K., Kim S.H. (2014). Inhibitory effect of galangin on atopic dermatitis-like skin lesions. Food Chem. Toxicol..

[34] Kwon D.J., Bae Y.S., Ju S.M., Goh A.R., Youn G.S., Choi S.Y., Park J. (2012). Casuarinin suppresses TARC/CCL17 and MDC/CCL22 production via blockade of NF-κB and STAT1 activation in HaCaT cells. Biochem. Biophys. Res. Commun..

[35] Gröne A. (2002). Keratinocytes and cytokines. Vet. Immunol. Immunopathol..

[36] Brandt E.B., Sivaprasad U. (2011). Th2 cytokines and atopic dermatitis. J. Clin. Cell. Immunol..

[37] Madonna S., Scarponi C., De Pità O., Albanesi C. (2008). Suppressor of cytokine signaling 1 inhibits IFN-γ inflammatory signaling in human keratinocytes by sustaining ERK1/2 activation. FASEB J..

[38] Kong L., Liu J., Wang J., Luo Q., Zhang H., Liu B., Xu F., Pang Q., Liu Y., Dong J. (2015). Icariin inhibits TNF-α/IFN-γ induced inflammatory response via inhibition of the substance P and p38-MAPK signaling pathway in human keratinocytes. Int. Immunopharmacol..

[39] Mitterman I., Aichberger K.J., Bünder R., Mothes N., Renz H., Valenta R. (2004). Autoimmunity and atopic dermatitis. Curr. Opin. Allergy Clin. Immunol..

[40] Vestergaard C., Kirstejn N., Gesser B., Mortensen J.T., Matsushima K., Larsen C.G. (2001). IL-10 augments the IFN-γ and TNF-α induced TARC production in HaCaT cells: A possible mechanism in the inflammatory reaction of atopic dermatitis. J. Dermatol. Sci..

[41] Kim J.E., Kim J.S., Cho D.H., Park H.J. (2016). Molecular mechanisms of cutaneous inflammatory disorder: Atopic dermatitis. Int. J. Mol. Sci..

[42] Nakazato J., Kishida M., Kuroiwa R., Fujiwara J., Shimoda M., Shinomiya N. (2008). Serum levels of Th2 chemokines, CCL17, CCL22, and CCL27, were the important markers of severity in infantile atopic dermatitis. Pediatr. Allergy Immunol..

[43] Nickel R.G., Casolaro V., Wahn U., Beyer K., Barnes K.C., Plunkett B.S., Freidhoff L.R., Sengler C., Plitt J.R., Schleimer R.P. (2000). Atopic dermatitis is associated with a functional mutation in the promoter of the C-C chemokine RANTES. J. Immunol..

[44] Rafatpanah H., Bennett E., Pravica V., McCoy M.J., David T.J., Hutchinson I.V., Arkwright P.D. (2003). Association between novel GM-CSF gene polymorphisms and the frequency and severity of atopic dermatitis. J. Allergy Clin. Immunol..

[45] Ackermann L., Harvima I.T. (1998). Mast cells of psoriatic and atopic dermatitis skin are positive for TNF-α and their degranulation is associated with expression of ICAM-1 in the epidermis. Arch. Dermatol. Res..

[46] Candi E., Schmidt R., Melino G. (2005). The cornified envelope: A model of cell death in the skin. Nat. Rev. Mol. Cell Biol..

[47] Proksch E., Brandner J.M., Jensen J.M. (2008). The skin: An indispensable barrier. Exp. Dermatol..

[48] Thyssen J.P., Kezic S. (2014). Causes of epidermal filaggrin reduction and their role in the pathogenesis of atopic dermatitis. J. Allergy Clin. Immunol..

[49] Scott I.R., Harding C.R. (1986). Filaggrin breakdown to water binding compounds during development of the rat stratum corneum is controlled by the water activity of the environment. Dev. Biol..

[50] Kezic S., Kammeyer A., Calkoen F., Fluhr J.W., Bos J.D. (2009). Natural moisturizing factor components in the stratum corneum as biomarkers of filaggrin genotype: Evaluation of minimally invasive methods. Br. J. Dermatol..

[51] Leung D.Y.M., Boguniewicz M., Howell M.D., Nomura I., Hamid Q.A. (2004). New insights into atopic dermatitis find the latest version: Science in medicine new insights into atopic dermatitis. J. Clin. Investig..

[52] Cho J.W., Lee K.S., Kim C.W. (2007). Curcumin attenuates the expression of IL-1β, IL-6, and TNF-α as well as cyclin E in TNF-α-treated HaCaT cells; NF-κB and MAPKs as potential upstream targets. Int. J. Mol. Med..

[53] Subhan F., Kang H.Y., Lim Y., Ikram M., Baek S.Y., Jin S., Jeong Y.H., Kwak J.Y., Yoon S. (2017). Fish scale collagen peptides protect against CoCl2/TNF- α-Induced cytotoxicity and inflammation via inhibition of ROS, MAPK, and NF-κB pathways in HaCaT cells. Oxid. Med. Cell. Longev..

